# Structural, Thermal Behaviour and Tribological Performance in Cold Rolling of Mineral Lubricants with Graphene Nanoplatelets Functionalized with Oleic Acid

**DOI:** 10.3390/nano16080495

**Published:** 2026-04-21

**Authors:** Batuhan Özakın, Kürşat Gültekin

**Affiliations:** 1 Department of Mechanical Engineering, Faculty of Engineering and Natural Sciences, Samsun University, Samsun 55420, Türkiye; 2 Department of Mechanical Engineering, Faculty of Engineering, Ondokuz Mayıs University, Samsun 55200, Türkiye; kursat.gultekin@omu.edu.tr

**Keywords:** graphene nanoplatelets, nanolubricants, cold rolling, tribology, dispersion stability

## Abstract

In this study, nanolubricants based on SAE 5W-30 mineral oil were formulated using oleic acid-functionalized graphene nanoplatelets (GNPs), and their colloidal stability, rheological behaviour, thermal stability, and tribological performance under cold rolling conditions were systematically investigated. The nanolubricants were prepared at GNP concentrations of 0.05, 0.1, 0.2, 0.4, and 0.6 wt%. FT-IR analysis confirmed successful functionalization, evidenced by the characteristic C=O band at approximately 1710 cm^−1^ and changes in CH_2_ stretching vibrations in the 2850–3000 cm^−1^ range. UV–VIS results indicated initially homogeneous dispersions; however, after three days, relative concentrations decreased to 95%, 90%, and 75% for 0.05, 0.2, and 0.6 wt% GNPs, respectively. Viscosity measurements showed minimal variation at low concentrations, with only a 0.64% increase at 0.2 wt% compared to the base oil. TGA revealed enhanced oxidative stability at low GNP contents, with the oxidation onset temperature increasing from 205.3 °C to 207.2 °C at 0.05 wt%, while a marked decline was observed at higher concentrations (176.8 °C at 0.6 wt%). In cold rolling experiments at a 3% reduction ratio, the rolling force was measured at 1341 N/mm with the neat lubricant, decreasing to 1210 N/mm with a lubricant containing 0.1 wt% GNPs, corresponding to an approximate 10% reduction. Compared with dry conditions, this reduction was approximately 21%. Surface roughness and 3D topography analyses further showed that GNPs-containing lubricants reduced asperities and promoted the formation of a more uniform tribofilm. At low concentrations, the improved lubrication performance of oleic acid-functionalized graphene nanoplatelets is attributed to their homogeneous dispersion in mineral oil, where physically adsorbed oleic acid improves colloidal stability by reducing agglomeration and promotes the formation of a stable tribofilm, facilitating interlayer sliding under boundary lubrication conditions. Overall, the findings demonstrate that oleic acid-functionalized GNPs, when used at optimal concentrations, significantly enhance both lubricant stability and cold rolling performance.

## 1. Introduction

Increasing energy efficiency is directly related to reducing friction and wear losses in mechanical systems [1]. It has been widely reported in the literature that nearly one-third of the total energy produced globally is dissipated due to friction and wear [2]. In this context, enhancing the performance of lubricants used in applications characterised by severe contact and high-load conditions, such as engines and metal-forming processes, has emerged as a critical research focus from both economic and environmental standpoints [3].

Although conventional mineral-based oils provide adequate lubrication performance under certain operating conditions, they may become insufficient as contact pressures and temperatures increase and as stringent environmental regulations are implemented [4]. In recent years, nanomaterial-based strategies have emerged as a key focus in the development of advanced lubricant additives to address these limitations [5]. Owing to their high specific surface areas, their ability to form protective tribofilms on contact surfaces, and their ability to mitigate friction and wear via multiple micro- and nanoscale mechanisms, nanostructured additives can exhibit superior performance compared to conventional additives [6]. In this context, numerous nanoparticles with diverse chemical compositions and morphologies have been investigated in the literature. Metal (e.g., Cu, Zn) and metal oxide nanoparticles (e.g., ZnO, TiO_2_, Al_2_O_3_, SiO_2_) enhance wear resistance through their high load-carrying capacity and surface-filling (mending effect) mechanisms [7,8]. Moreover, sulfide nanoparticles (e.g., MoS_2_, WS_2_) and layered inorganic materials (e.g., h-BN, phyllosilicate mineral powders) act as solid lubricants, with their low shear strength and lamellar crystal structures enabling substantial reductions in friction [9,10,11]. Conversely, carbon-based nanomaterials (e.g., graphite, carbon nanotubes, fullerenes, and graphene derivatives) offer notable benefits for tribological applications owing to their low density, excellent chemical stability, and hybridised structures that confer atomic-scale lubricity [12]. Notably, two-dimensional graphene and graphene oxide exhibit reduced friction and wear owing to their extensive contact area, exceptional mechanical strength, and lamellar morphology that promotes facile sliding between surfaces. Furthermore, graphene’s high thermal conductivity enhances heat dissipation at the contact interface, thereby improving the lubricant’s thermal stability [13]. One of the main challenges in effectively utilizing graphene nanoparticles in lubricant systems is their tendency to agglomerate and settle over time due to high surface energies. This not only compromises the rheological stability of the lubricant but also limits the sustainability of the expected tribological improvements [14]. Surface functionalization of nanoparticles is a key strategy for improving dispersion stability in lubricant systems [15]. In the literature, surface functionalization of graphene and the use of appropriate surfactants have been widely reported as effective strategies to enhance dispersion stability in lubricant media. For example, Mungse et al. [16] reported that graphene oxide (GO) and reduced graphene oxide (rGO) functionalized with octadecylsilane exhibited high dispersion stability in oil-based fluids, and that the resulting nanolubricants reduced friction by 37% and wear by 17%. Chouhan et al. [17] reported that incorporating aminoborate-functionalized reduced graphene oxide into aqueous lubricants provided long-term dispersion stability as an environmentally friendly, scalable additive, while increasing thermal conductivity by 68%. Furthermore, at a concentration of 0.2 wt%, friction and wear were reduced by 70% and 68%, respectively. Mousavi et al. [18] investigated the performance of an environmentally friendly, synthetic biodegradable polyalphaolefin (PAO) based oil by incorporating 0.3 wt% Cu/TiO_2_/MnO_2_-doped graphene oxide under a wide range of temperature and operating conditions; the results showed improvements in viscosity index, pour point, and flash point, along with up to a 46% enhancement in wear resistance. La et al. [19] demonstrated that graphene nanoplatelets (GNPs) modified sequentially with a surfactant and oleic acid achieved stable dispersion in oil. At a low addition level of 0.05 wt%, the modified GNPs reduced the wear scar diameter on steel surfaces by 35%, highlighting their effectiveness in enhancing tribological performance. Wu et al. [20] functionalized graphene with octadecylamine and dicyclohexylcarbodiimide and incorporated it into base oil with the aid of a dispersant. The resulting lubricant exhibited not only high dispersion stability but also enhanced tribological performance, reducing the coefficient of friction by approximately 44% and wear scar depth by 90%. These studies indicate that surface modifications strengthen the interaction between graphene and the oil matrix, thereby improving colloidal stability. Moreover, because graphene nanosheets are present at very low concentrations in mineral oil, their tribological performance is strongly influenced by their interactions with the surrounding lubricant. Surface modification with oleic acid enhances the compatibility of graphene nanosheets with mineral oil, thereby improving their dispersion stability. This leads to the formation of an interfacial adsorption layer or tribofilm at the contact interface, which facilitates shear accommodation and reduces direct metal-to-metal contact. However, a significant portion of existing research evaluates tribological performance using standard tests, such as pin-on-disk or four-ball setups [21,22]. Nevertheless, these studies have largely focused on standard tribological tests, with limited attention to metal forming applications that reflect actual manufacturing processes. In contrast, processes such as cold rolling, which are characterised by high contact pressures and intense surface interactions, provide a more representative platform for evaluating the real-world performance of developed nanolubricants [23]. Moreover, a comprehensive assessment of graphene-containing mineral oils should encompass not only their frictional performance but also key factors such as dispersion stability, viscosity variation, and thermal and oxidative stability.

In this study, nanolubricant systems based on SAE 5W-30 mineral oil were developed using GNPs surface-modified with oleic acid, and their colloidal, rheological, thermal, and tribological properties were investigated in detail. Furthermore, the tribological performance of the developed nanolubricants was evaluated beyond laboratory-scale tests under actual cold rolling conditions. This approach provides a comprehensive assessment of graphene-enhanced mineral oils, addressing both their fundamental physicochemical properties and their industrial applicability. SAE 5W-30 mineral oil was employed as a model base lubricant to systematically investigate the effect of GNPs under controlled conditions. While it does not represent a commercial cold rolling fluid, it provides a simplified and reproducible platform to isolate nanoparticle-induced effects. Therefore, the results should be interpreted as a fundamental assessment of tribological behaviour rather than a direct industrial formulation study.

## 2. Materials and Methods

### 2.1. Materials

In the experimental work, SAE 5W-30 mineral oil (Petro Time), graphene nanoplatelets (GNPs) with an average thickness of 5 nm and a diameter of 30 µm (Nanografi Inc., Ankara, Türkiye), oleic acid (Bilim Chemistry Inc., İstanbul, Türkiye), cyclohexane (Merck, Darmstatd, Germany), and other auxiliary chemicals were used. All materials were commercially obtained and evaluated in their as-received form during the experiments.

### 2.2. Functionalization of GNPs with Oleic Acid and Preparation of Nanolubricants

**Figure 1:**
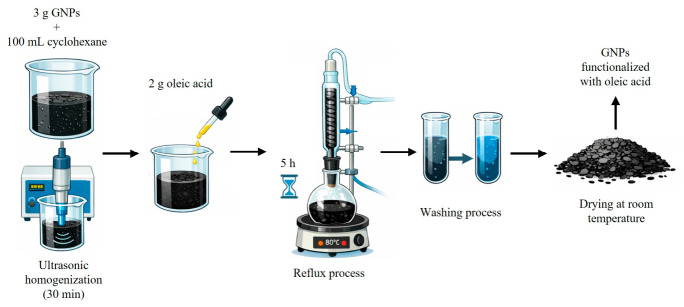
Oleic acid functionalization of GNPs.

Three grams of GNPs were dispersed in 100 mL of cyclohexane and ultrasonicated for 30 min to ensure uniform distribution. Subsequently, 2 g of oleic acid was added, and the mixture was stirred under reflux at 80 °C for 5 h. Following reflux, the resulting precipitate was collected and washed three times with cyclohexane to remove excess oleic acid and physically unbound species. The final product was then dried at room temperature [24,25]. A schematic illustration of the oleic acid functionalization of GNPs is presented in Figure 1.

It should be noted that the amount of oleic acid associated with the GNP surface was not quantitatively determined, as the modification is primarily based on non-covalent adsorption. Therefore, the effectiveness of the functionalization is evaluated through qualitative characterisation (FT-IR) and performance-based analyses, including dispersion stability and tribological behaviour.

**Figure 2:**
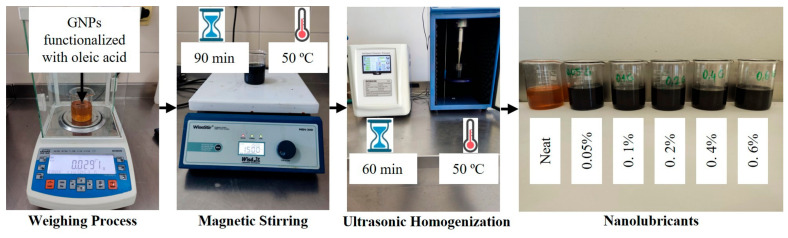
Preparation process of the nanolubricants.

Oleic acid-functionalized GNPs were incorporated into SAE 5W-30 mineral oil at five different weight fractions (0.05, 0.1, 0.2, 0.4, and 0.6 wt%). The resulting lubricant mixtures were stirred at 50 °C and 1500 rpm on a magnetic stirrer for 90 min to ensure homogeneous dispersion of the GNPs in the oil. This was followed by ultrasonication for 60 min to homogenise the nanolubricants further [26]. The preparation procedure of the nanolubricants is illustrated in Figure 2.

### 2.3. Methods

#### 2.3.1. Fourier Transform Infrared (FT-IR) Spectroscopy

The chemical bonding and functional groups of the samples were analysed using Fourier transform infrared (FT-IR) spectroscopy. FT-IR spectra were recorded over 650–4000 cm^−1^ to evaluate characteristic functional group vibrations. By comparing the spectra of modified and unmodified samples, the effect of surface functionalization on the chemical structure was assessed.

#### 2.3.2. Ultraviolet–Visible (UV–VIS) Spectroscopy

The dispersion stability of the nanolubricant systems was evaluated by UV–VIS spectrophotometry. Based on the Beer–Lambert law, which relates the absorbance to the concentration of the dispersed phase, the lubricant samples were diluted 50-fold with ethanol before measurement [27]. Absorbance values of the prepared nanolubricants were recorded immediately after sample preparation and after three days of storage to assess dispersion stability over time. Measurements at 400 nm were used to calculate relative concentrations. The selection of 400 nm, instead of the characteristic π–π* transition region (approximately 270 nm) of graphene, was intentional, as the primary aim of the UV–VIS analysis was to monitor dispersion stability rather than electronic structure. At this wavelength, the influence of intrinsic absorption peaks is minimized, and the measurements become more sensitive to scattering effects associated with suspended nanoparticles, allowing a more reliable evaluation of dispersion stability over time. The initial absorbance was used as a reference and set to 100 units, and relative concentration values were determined by comparing the absorbance measured after 3 days with the initial value [28]. The resulting relative concentration data were analysed comparatively to evaluate the dispersion stability of the nanolubricants quantitatively. While long-term stability tests (e.g., ≥30 days) would provide additional information under static conditions, the present study focuses on dynamic lubrication environments, where continuous shear can partially mitigate sedimentation effects.

#### 2.3.3. Viscosity Measurements

The dynamic viscosity of SAE 5W-30 mineral oil containing oleic acid-functionalized GNPs was measured using a rotational viscometer. Before measurement, the lubricant samples were equilibrated to room temperature, and an appropriate volume was transferred to the measurement chamber. The tests were conducted at a shear rate of 250 s^−1^, and the instrument directly recorded the corresponding viscosities.

#### 2.3.4. Thermogravimetric Analysis (TGA)

The thermal decomposition behaviour and mass-loss characteristics of the samples were investigated by thermogravimetric analysis (TGA) under air. During the analysis, the samples were heated from room temperature to 800 °C at 10 °C/min under a continuous flow of air, and the temperature-dependent mass changes were continuously recorded [29]. Conducting TGA under an air atmosphere enabled the evaluation of the oxidative degradation behaviour and thermal stability of the samples.

#### 2.3.5. Rolling Tests

To evaluate the tribological behaviour of the lubricants, experiments were conducted on a cold rolling setup equipped with specially roughened rollers. Cold rolling experiments were conducted on a single-stand mill operating at 10 rpm using low-carbon mild steel (DC06 grade) specimens with excellent formability, each having dimensions of 200 × 30 × 1 mm^3^. During the rolling process, two different reduction ratios (3% and 9%) were applied. The selected reduction ratios, along with roughened rolls and a single-pass configuration, were intentionally adopted to ensure controlled and reliable tribological evaluation. The higher reductions were limited by material instability and machine constraints. The surface roughness of the rollers was determined from measurements taken at different locations of the upper and lower rolls, yielding an arithmetic average roughness (R_a_) of 3.4 µm. Before each rolling test, 2 g/m^2^ of the developed lubricant sample was applied to the roller surfaces [30]. The rolling forces generated during the tests were continuously monitored and recorded by the system’s load cell. The reported rolling force values correspond to the average of repeated measurements performed under identical conditions. To explicitly demonstrate experimental variability, the standard deviations were calculated and shown as error bars in the relevant figures. The rolling test parameters and experimental notation are summarised in Table 1.

The surface roughness of the samples was measured using a contact-type profilometer with a needle tip. Measurements were performed with a cut-off length of 0.8 mm and an evaluation length of 2.5 mm, and the arithmetic average roughness (R_a_) was calculated to characterise the surface. At least three measurements were taken at different locations on each sample, and the results were reported as average values. Three-dimensional surface topography of the samples was analysed using a non-contact optical 3D profilometer, and the surface morphology was evaluated based on data obtained from the scanned areas. The selected conditions represent the optimal cases, enabling a clear interpretation of the lubrication mechanism without redundancy.

## 3. Results and Discussion

### 3.1. Fourier Transform Infrared (FT-IR) Spectroscopy Results

**Figure 3:**
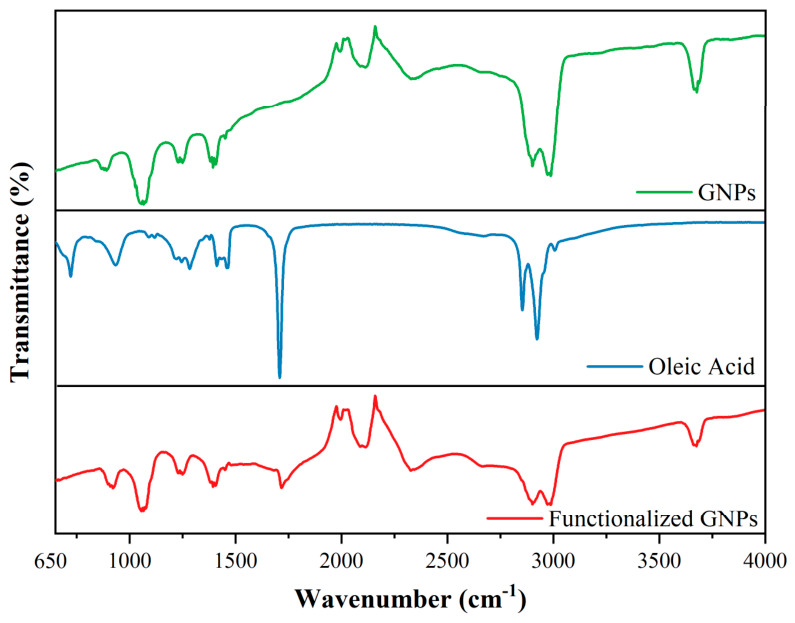
FT-IR spectra of GNPs and oleic acid-functionalized GNPs.

The FT-IR spectra of GNPs and oleic acid-functionalized GNPs are presented in Figure 3. The absence of a broad band in the 3300–3450 cm^−1^ region suggests that hydrogen-bonded hydroxyl groups are not dominant in the sample. The weak band observed around approximately 3750 cm^−1^ is attributed to free or weakly hydrogen-bonded –OH stretching vibrations, which may originate from adsorbed moisture or residual surface species. Bands appearing between 2850–3000 cm^−1^ are attributed to the symmetric and asymmetric stretching vibrations of CH_2_ groups. The band observed at approximately 1380 cm^−1^ corresponds to C–OH stretching vibrations. Bands in the 1220–1260 cm^−1^ and 1050–1120 cm^−1^ ranges are assigned to alkoxy C–O bonds. Finally, bands appearing in the 870–900 cm^−1^ region correspond to out-of-plane C–H vibrations of aromatic rings [31,32,33,34]. It should be noted that commercially available graphene nanoplatelets may contain minor oxygen-containing functional groups due to their production process. Moreover, oleic acid functionalization introduces additional oxygen-containing bonds, as reflected in the FT-IR spectra. Therefore, the observed bands should not be interpreted as evidence of graphene oxide, but rather as confirmation of surface functionalization. The main FT-IR bands of oleic acid are observed at 2923–2854 cm^−1^, corresponding to aliphatic –CH_2_ stretching vibrations; at 1459–1412 cm^−1^, corresponding to CH_3_/CH_2_ bending vibrations; and at 1284 cm^−1^, assigned to C–O stretching. The FT-IR peak observed at 1710 cm^−1^ corresponds to the C=O stretching vibration of carboxylic acid groups from oleic acid. This indicates surface interaction rather than covalent ester bond formation. Therefore, the modification mechanism is attributed to non-covalent adsorption of oleic acid onto the GNP surface [29,35]. In the FT-IR spectra of oleic acid-functionalized GNPs, the characteristic bands of GNPs are generally preserved. The band observed at 1710 cm^−1^ corresponds to a distinctive vibration characteristic of oleic acid. Moreover, changes in the intensity of the bands in the 2850–3000 cm^−1^ range, which are attributed to the symmetric and asymmetric CH_2_ stretching vibrations of oleic acid, indicate the successful functionalization of GNPs with oleic acid.

**Figure 4:**
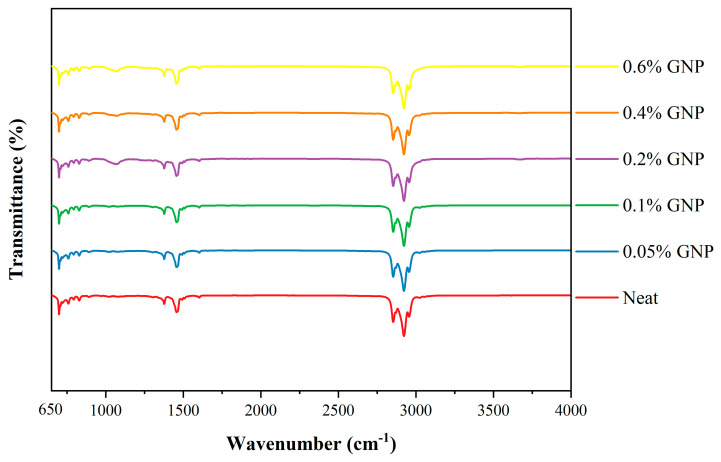
FT-IR spectra of neat oil and GNPs-containing lubricants.

Figure 4 presents the FT-IR spectra of the neat 5W-30 mineral oil and the GNPs-containing lubricants. The characteristic bands of the lubricants are as follows: the bands observed at 2854, 2921, and 2958 cm^−1^ are attributed to the aliphatic C–H stretching vibrations of the main oil backbone. The band at 1376 cm^−1^ corresponds to the symmetric bending vibrations of CH_3_ groups, while the band at 1460 cm^−1^ is assigned to the bending vibrations of CH_2_ groups. Finally, the band observed at 699 cm^−1^ is attributed to the out-of-plane C–H bending vibrations of the phenyl ring [36,37,38]. It is noteworthy that the FT-IR bands of the GNPs-containing lubricants exhibit patterns similar to those of the neat oil. This observation indicates that the lubricants maintain chemical stability and that the graphene nanoplatelets are physically dispersed within the oil matrix [39].

### 3.2. Ultraviolet–Visible (UV–VIS) Spectroscopy Results

**Figure 5:**
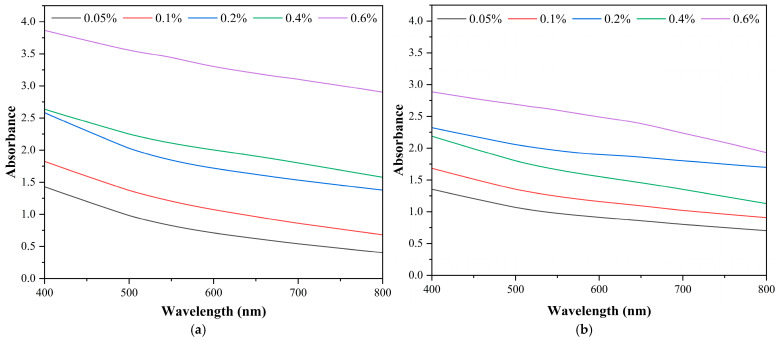
UV–VIS spectra of GNPs-containing mineral lubricants: (**a**) Day 0, (**b**) Day 3.

The UV–VIS spectra obtained to evaluate the dispersion stability of GNPs-containing mineral lubricants are presented in Figure 5. It should be noted that the UV–VIS analysis in this study is based on a relative absorbance approach to evaluate dispersion stability over time, rather than absolute concentration determination. Therefore, no calibration curve was established, and the results should be interpreted in terms of comparative stability behaviour. Figure 5a shows the spectra measured immediately after nanolubricant preparation. The absorbance values increase systematically with increasing GNP concentration, indicating a direct correlation between absorbance and particle concentration. This indicates that the GNPs were well-dispersed at the initial stage, achieving homogeneous dispersion stability. Therefore, immediately after preparation, the nanolubricants exhibited successful dispersion, and Beer–Lambert law behaviour was maintained [40].

Figure 5b shows the UV–VIS spectra of the nanolubricants after three days of storage. A comparison with the spectra in Figure 5a reveals a decrease in absorbance across all concentrations, indicating a reduction in the number of dispersed particles and the onset of sedimentation. Notably, the decrease is less pronounced at lower concentrations (0.05 and 0.1 wt%) and more significant at higher concentrations (0.4 and 0.6 wt%). This behaviour can be attributed to stronger Van der Waals interactions, higher collision frequency, and accelerated agglomeration at elevated nanoparticle concentrations, leading to faster sedimentation [41]. To quantitatively and comparatively assess time-dependent changes in UV–VIS absorbance, absorbance values were normalised to the initial measurement, and relative concentration plots were analysed to evaluate dispersion stability. It should be noted that the 1:50 dilution of the samples before UV–VIS analysis partially weakened the colloidal equilibrium, increasing interparticle distances and reducing the influence of the lubricant’s inherent viscosity. Consequently, sedimentation and agglomeration processes occurred more rapidly than in the undiluted oil matrix, and the observed stability loss manifested over a shorter timescale. Therefore, the three-day UV–VIS evaluation provides an accelerated indication of the longer-term stabilisation behaviour expected under practical lubricant conditions.

**Figure 6:**
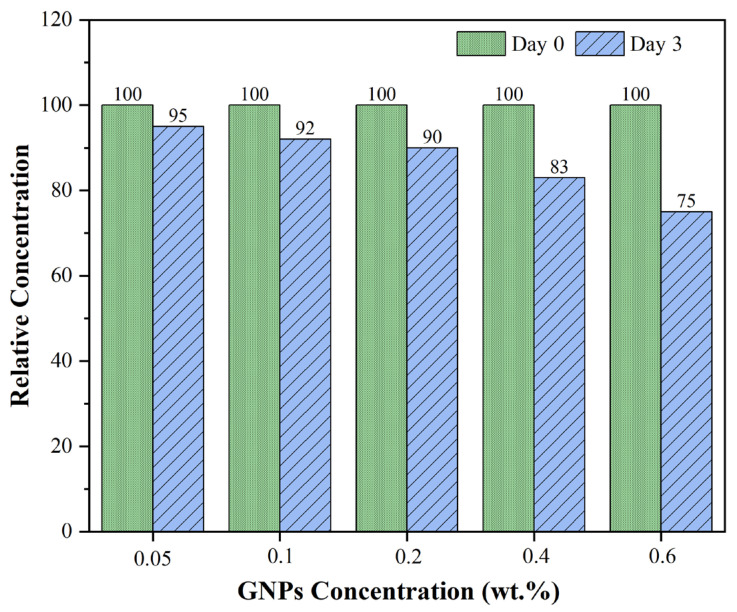
Relative concentration profiles of GNPs-containing mineral lubricants.

For a quantitative evaluation of the UV–VIS results, the relative concentration profiles of the GNPs-containing mineral lubricants, directly reflecting the previously observed spectral trends, are presented in Figure 6. All samples were initially normalised to 100% to establish a reference point. After three days, the relative concentrations decreased to 95% for the 0.05 wt% GNPs lubricant, 90% for the 0.2 wt% GNPs lubricant, and 75% for the 0.6 wt% GNPs lubricant. These results confirm that the observed reduction in relative concentration over time corresponds to an effective loss of nanoparticles from the system due to sedimentation and agglomeration. Another notable observation is that dispersion stability decreases with increasing GNPs concentration. At higher GNPs loadings, stronger interparticle interactions and increased collision frequency accelerate agglomeration kinetics, leading to more pronounced sedimentation [42]. From an engineering perspective, at low to moderate GNPs concentrations (0.05–0.2 wt%), relative concentrations above 90% are maintained, indicating a more stable dispersion. In contrast, at higher concentrations, significant phase separation occurs rapidly. Therefore, an optimal additive concentration can be identified to balance lubricant performance and dispersion stability.

### 3.3. Viscosity Results

**Figure 7:**
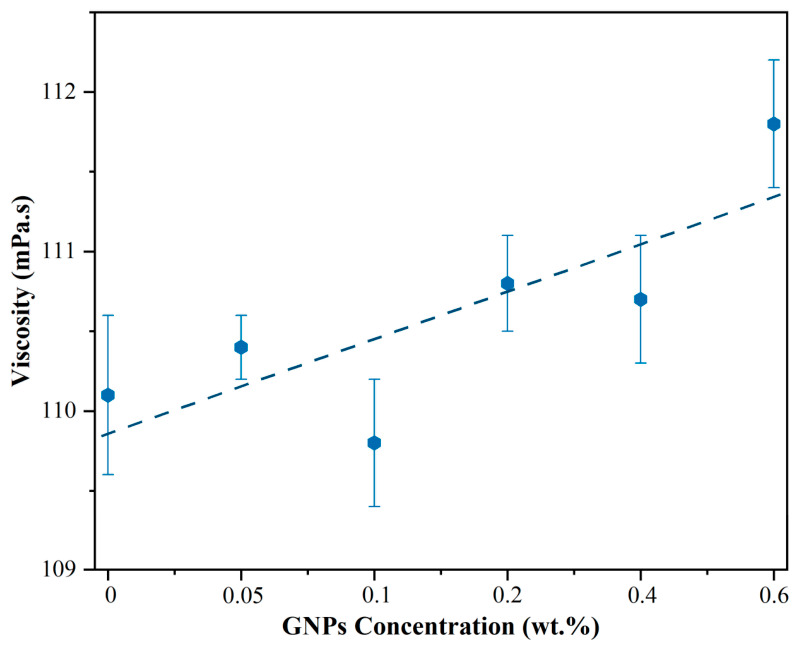
Relationship between GNPs concentration and viscosity changes.

Figure 7 illustrates the relationship between GNPs concentration and viscosity in 5W-30 mineral oil. The plot shows a general trend of increasing viscosity with rising GNPs content. At low concentrations (0.05–0.2 wt%), the changes are minimal, with the viscosity of the 0.2 wt% GNPs-containing lubricant increasing by only 0.64% compared to the base oil. This minimal effect is attributed to the sparse distribution of GNPs in the oil, acting as isolated solid obstacles, resulting in negligible or slightly reduced viscosity at low loadings. In contrast, at higher concentrations (≥0.4 wt%), the viscosity increase becomes more pronounced. This is due to the solid fraction and particle geometry of GNPs, which enhance the effective surface area and interparticle interactions, thereby increasing flow resistance and contributing to the observed viscosity rise [43]. The minimal viscosity variation at low concentrations indicates that the addition of GNPs does not significantly alter the base oil’s flow characteristics under the tested conditions, which is advantageous for industrial applications.

### 3.4. Thermogravimetric Analysis (TGA) Results

**Figure 8:**
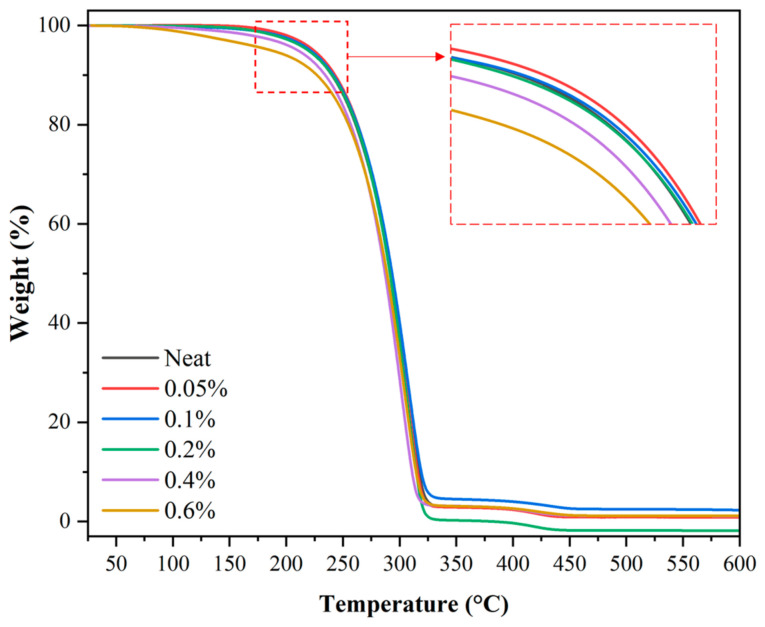
TGA curves showing the temperature-dependent weight change in mineral oil-based lubricants as a function of GNPs concentration.

Thermogravimetric analysis (TGA) was conducted to investigate the effect of GNPs content on the thermal degradation behaviour. Figure 8 presents the TGA curves showing the weight percentage as a function of temperature.

The thermal behaviour exhibits noticeable changes with varying GNPs concentration. For all samples, the weight loss in the 25–175 °C range is negligible, indicating thermal stability in this region. As the GNPs content increases, a slight shift in the degradation onset temperature and a more gradual weight loss at higher temperatures are observed. At low concentrations (0.05–0.1 wt%), the curves are very similar, whereas at higher concentrations (0.2–0.6 wt%), the weight loss at elevated temperatures occurs earlier and is more pronounced. Specifically, samples containing 0.4 wt% and 0.6 wt% GNPs exhibit lower residual mass at high temperatures and faster overall degradation than the pristine sample. In contrast, the differences between low and medium loadings (0.05–0.1 wt%) are relatively limited. Overall, as the GNPs concentration increases, the divergence between the curves becomes more pronounced, indicating that the thermal degradation profile depends on the additive content. Oxidation onset temperatures are particularly important for a more precise quantitative assessment of these effects.

The oxidation onset temperatures (Tₒ) of the mineral oil-based lubricants as a function of GNPs concentration are presented in Table 2. The obtained Tₒ values indicate a nonlinear increase in the system’s oxidative thermal stability with increasing GNPs content. The neat base oil exhibited a Tₒ of 205.3 °C, which increased to 207.2 °C and 206.4 °C at 0.05 wt% and 0.1 wt% GNPs, respectively. However, a decrease was observed at higher concentrations, with the Tₒ dropping to 176.8 °C at 0.6 wt% GNPs. These results suggest the presence of an optimum additive concentration for enhancing oxidative thermal stability. The increase in Tₒ at low concentrations (0.05–0.1 wt%) can be attributed to barrier and stabilisation mechanisms commonly reported in nanolubricant systems. Well-dispersed nanoparticles hinder oxygen diffusion within the lubricant, prolonging the path for oxidative reactions and delaying the kinetic conditions necessary for oxidation initiation. Additionally, the reduction in chain mobility at the lubricant–additive interface, due to improved interfacial compatibility, slows free-radical formation and oxidative chain scission. Furthermore, carbon-based additives generally exhibit high thermal conductivity, which, when uniformly distributed at low concentrations, can mitigate local temperature rises and enable more controlled thermal degradation [44,45,46]. Considering these mechanisms together, the observed ~2 °C increase in Tₒ at 0.05 wt% GNPs can be attributed primarily to the physical barrier effect and interfacial stabilisation. The limited magnitude of this increase suggests that the base lubricant already possesses a certain degree of thermal stability and that the additive does not act as a radical-scavenging chemical stabiliser; the effect is largely physical in nature. At 0.2 wt% GNPs, the decrease in Tₒ to 200.1 °C indicates that a structural threshold in the system has been surpassed. Beyond this point, microvoid formation in the interfacial regions becomes possible, providing alternative diffusion pathways for oxygen and accelerating the kinetics of oxidative reactions. The sharp decrease in Tₒ observed at 0.4 wt% and, particularly, at 0.6 wt% indicates that stabilisation mechanisms are no longer dominant and that degradation-promoting factors are taking effect. Several mechanisms can explain this: at high concentrations, maintaining a homogeneous dispersion becomes challenging; thermal accumulation within agglomerates accelerates the onset of oxidation; weak interfacial bonding promotes radical formation; and, at high additive loadings, accelerated peroxide decomposition can further generate free radicals, initiating oxidative chain reactions [47,48]. The approximately 28 °C decrease in Tₒ at 0.6 wt% clearly indicates a significant weakening of the system’s oxidative stability. This suggests that at high GNPs loadings, the additive may act as a potential degradation-promoting factor rather than a stabiliser.

### 3.5. Rolling Results and Surface Characterisation

**Figure 9:**
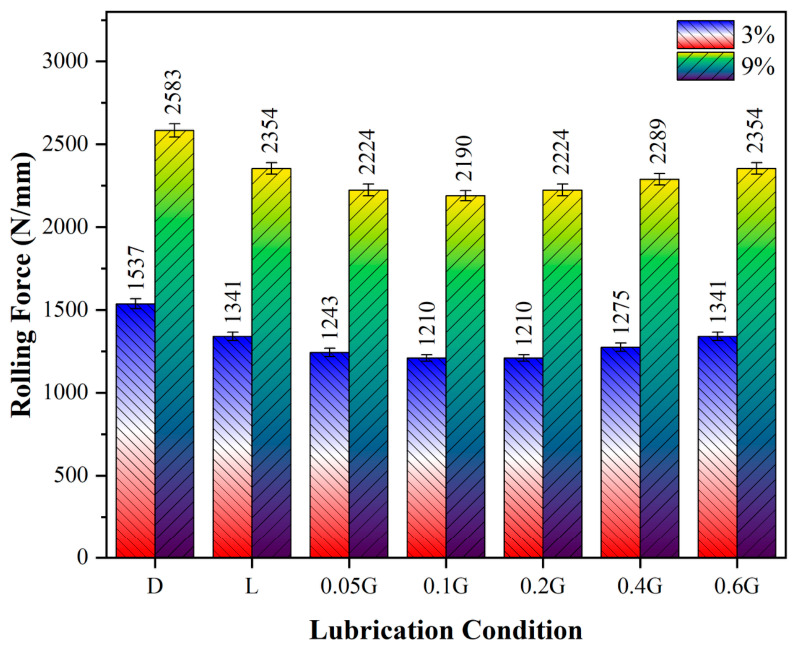
Rolling forces measured during rolling tests under different lubrication conditions with GNPs-lubricated mineral oils.

Figure 9 shows the rolling forces corresponding to the lubrication conditions of the GNPs-enhanced mineral lubricants. It is noteworthy that rolling forces increase as the reduction ratio decreases. The error bars shown in Figure 9 represent the standard deviations of the measurements. The relatively small deviations indicate good repeatability and consistency of the experimental results. For instance, when the base lubricant is used, the rolling force is 1341 N/mm at a 3% reduction ratio and rises to 2354 N/mm at a 9% reduction ratio. A similar trend is consistently observed under other experimental conditions. The main reasons for this increase are the greater plastic deformation, the enlarged roller–material contact area, and the associated rise in the friction coefficient. When evaluating the effect of GNPs content, the rolling forces at a 3% reduction ratio for mineral lubricants with 0.05%, 0.1%, and 0.2% wt. GNPs are 1243, 1210, and 1210 N/mm, respectively. For the same GNPs concentrations at a 9% reduction ratio, the rolling forces are 2224, 2190, and 2224 N/mm, respectively. This indicates that the most effective reduction in rolling force occurs at an optimal GNPs concentration. The experimental rolling force data suggest that this optimal concentration is approximately 0.1–0.2% wt. The observed improvement in GNPs-containing mineral lubricants can be associated with the lamellar (layered) crystal structure of GNPs. The low interlayer shear resistance facilitates easy shearing at the contact surfaces, potentially leading to the formation of a tribofilm, as reported in the literature. Additionally, the GNPs fill micro-voids and asperities on the surface, thereby regulating the actual contact area, reducing the friction coefficient, and contributing to a decrease in rolling force [49]. At higher GNPs concentrations of 0.4% and 0.6% wt., the rolling forces at a 3% reduction ratio are 1275 and 1341 N/mm, respectively, while at a 9% reduction ratio, they are 2289 and 2354 N/mm, respectively. These results indicate that exceeding the optimal GNPs concentration leads to a renewed increase in rolling forces. The primary reasons for this increase beyond the optimal concentration include GNPs’ tendency to agglomerate, enhanced interparticle interactions, and the resulting reduction in fluidity. Moreover, at high concentrations, the loss of homogeneous dispersion can lead to localised clusters of hard particles in the contact zone, potentially causing an abrasive effect [50]. Another noteworthy observation is the interaction between the reduction ratio and the effect of GNPs. Under rolling conditions with a 3% reduction ratio using 0.1% wt. In GNPs-lubricated oil, the rolling force decreased by approximately 21% compared to dry conditions. At a 9% reduction ratio, rolling with GNPs-lubricated oil resulted in an approximately 15% reduction in rolling force relative to dry conditions. The observation of similar trends under other lubricant conditions indicates that the additive’s beneficial effect is more pronounced at lower reduction ratios. At higher reduction ratios, the increased plastic deformation, expanded contact area, and elevated normal pressures limit the relative improvement provided by the additive. These findings demonstrate that GNPs, when used at appropriate concentrations, can significantly reduce rolling forces. Consequently, industrially relevant benefits such as reduced energy consumption, extended roll life, and decreased roll wear can be achieved.

**Figure 10:**
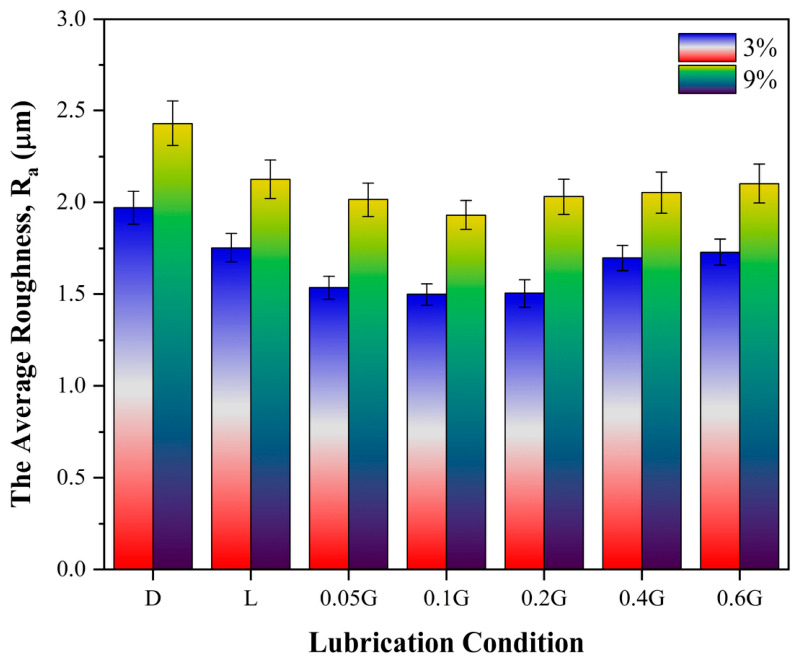
Average surface roughness (R_a_) results corresponding to lubrication conditions for mineral oil lubricants containing GNPs.

Figure 10 presents the average surface roughness (R_a_) results for mineral oils containing GNPs under different lubrication conditions. The figure clearly illustrates the effect of GNPs-lubricated oils on tribological behaviour during the rolling process, showing that the interaction between the reduction ratio and additive concentration is a key factor in determining surface quality. In all lubrication conditions, R_a_ values obtained at a 9% reduction ratio are higher than those at 3%, which can be attributed to increased contact length and pressure, resulting in a larger real contact area, greater asperity deformation, and approaching boundary lubrication conditions [51]. Under dry conditions, both the rolling force and surface roughness reach their maximum value due to high friction. While the use of base oil partially reduces metal–metal contact, it cannot eliminate asperity interactions. At a 3% reduction ratio, low to moderate GNPs concentrations (particularly 0.1–0.2 wt%) decrease the coefficient of friction due to the lamellar graphene structure’s easy interlayer sliding, its capacity to form a tribofilm on the surface, and its ability to fill microvoids, thereby improving surface topography. Consequently, significant reductions in both rolling force and R_a_ are observed. These conditions indicate that the system operates in a mixed lubrication regime, with a relatively stable oil film and more homogeneous sliding. However, when the reduction ratio increases to 9%, the rising contact pressure challenges tribofilm stability, reduces the oil film thickness, and brings the system closer to the boundary lubrication regime. As a result, the coefficient of friction increases, causing both the rolling force and surface roughness to rise again. Furthermore, the increase in R_a_ observed at higher additive concentrations (0.4–0.6 wt%) can be attributed to nanoplatelet agglomeration, increased effective viscosity, and possible third-body effects [52]. Therefore, when evaluated from both mechanical and tribological perspectives, it has been determined that the addition of GNPs improves surface quality and energy efficiency within an optimal concentration range primarily by controlling interfacial friction rather than altering the material’s yield strength. However, under conditions of high pressure and high additive concentration, its effectiveness diminishes.

**Figure 11:**
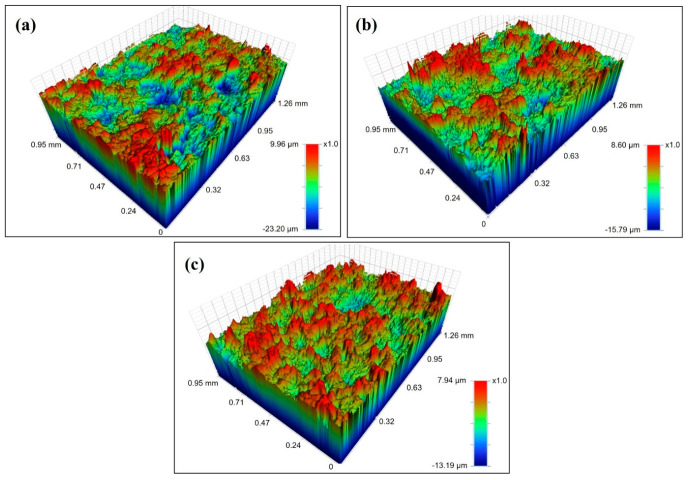
3D surface topography images obtained after the rolling process at a 3% reduction ratio under different lubrication conditions: (**a**) dry, (**b**) base oil without additives, and (**c**) lubricant containing 0.1 wt% GNPs.

Figure 11 shows the three-dimensional surface topographies obtained after the rolling process at a 3% reduction ratio. The images correspond to (a) dry condition, (b) base oil, and (c) 0.1 wt% GNPs-enhanced lubricant, respectively. A comparative analysis of surface topographies indicates that lubrication conditions significantly affect surface morphology and roughness. In Figure 11a, representing the dry-rolling condition, the surface topography appears highly irregular, with sharp peak–valley structures. The colour distribution shows high peaks in red and deep valleys in blue, spanning large surface areas. This indicates direct metal-to-metal contact during rolling, with high friction leading to intensified adhesive wear and localised microscale plastic deformation. Additionally, the pronounced peaks and deep depressions suggest active material transfer and wear mechanisms induced by friction during the rolling process [53]. The surface topography under the rolling condition with the base oil is shown in Figure 11b. In this case, the peak–valley distribution on the surface appears more uniform compared to the dry condition. The lubricant film forms a partial separating layer between the contact surfaces, reducing direct metal-to-metal contact and limiting frictional effects. As a result, the number of deep valleys and sharp peaks on the surface decreases. However, the surface morphology is not entirely smooth, and some regions still exhibit pronounced elevations. This indicates that base oils possess limited film strength under high-pressure plastic deformation conditions. The surface topography after rolling with the 0.1 wt% GNPs-enhanced lubricant is presented in Figure 11c. Here, the surface exhibits a more balanced morphology with a more regular peak–valley distribution. The height of sharp peaks is reduced, and the depth of valleys is more confined. This improvement can be attributed to the additive, which enhances lubricant film stability and provides more effective tribological protection within the contact zone. The additive particles form a load-bearing tribofilm between the contact surfaces, promoting more uniform plastic flow at the microscale while reducing direct asperity contact. Additionally, the additive likely contributes to a mending effect or micro-level polishing effect by partially filling surface irregularities [54,55]. Moreover, the consistency between the Ra results and the 3D surface topography observations further supports the validity of the asperity reduction behaviour.

The combined evaluation of the three surface topographies clearly demonstrates that the lubrication conditions play a decisive role in determining the post-rolling surface quality. Under dry conditions, high friction and direct metal-to-metal contact result in a more irregular and aggressive surface morphology, whereas the use of a lubricant significantly reduces this irregularity. In particular, the use of a lubricant containing additives facilitates a more controlled deformation of surface asperities, contributing to a more uniform surface topography. This indicates that such additives have significant potential to reduce friction, protect the surface, and improve surface quality during rolling processes.

The 3D surface topography images are presented as representative qualitative visualisations to support the quantitative surface roughness (R_a_) results. While additional areal surface parameters (e.g., S_a_, S_q_, S_sk_, S_ku_) could provide more detailed surface characterisation, the scope of this study focuses on comparative tribological performance. The consistency between R_a_ measurements and 3D topography observations confirms the validity of the observed asperity reduction and improved surface uniformity.

## 4. Conclusions

In this study, the structural, colloidal, thermal, and tribological properties of mineral oil-based nanolubricants containing oleic acid–functionalized GNPs were systematically investigated, and the following results were obtained:

1. FT-IR analyses confirmed the successful functionalization of GNPs with oleic acid, as evidenced by the C=O band observed around 1710 cm^−1^ and the changes in CH_2_ vibrations in the 2850–3000 cm^−1^ range.
2. UV–VIS results indicated that a homogeneous dispersion was initially achieved in all samples. After 3 days of storage, the relative concentrations were 95%, 90%, and 75% for 0.05%, 0.2%, and 0.6% GNPs, respectively, indicating a greater tendency toward agglomeration at higher concentrations.
3. Viscosity measurements showed that the flow properties were largely preserved at low GNPs concentrations. In the 0.2% GNPs sample, viscosity increased by only 0.64% compared to the base oil.
4. TGAs revealed that low GNPs concentrations did not significantly affect oxidative stability. The oxidation onset temperature of the base oil, 205.3 °C, increased to 207.2 °C at 0.05% GNPs, but decreased at higher concentrations, reaching 176.8 °C at 0.6% GNPs.
5. Cold rolling tests demonstrated that the addition of GNPs improved tribological performance. At a 3% reduction, the rolling force decreased from 1341 N/mm for the base oil to 1210 N/mm with 0.1% GNPs, representing approximately a 21% reduction compared to dry conditions.
6. Surface roughness and 3D topography analyses showed that a more uniform surface morphology and lower roughness values were achieved, particularly in the 0.1–0.2% GNPs range.
7. Overall, the 0.1–0.2% GNPs concentration range was found to provide optimal results for dispersion stability, thermal behaviour, and tribological performance. The developed nanolubricants have demonstrated the potential to reduce friction and energy losses in cold rolling processes.
8. From an industrial implementation perspective, several factors beyond laboratory-scale tribological performance should be considered. These include the cost-effectiveness of large-scale functionalization of graphene nanoplatelets, long-term oxidative and colloidal stability under continuous industrial recirculation, and compatibility with filtration systems used in cold-rolling mills. In addition, the present results indicate that at higher GNP concentrations (0.4–0.6 wt%), agglomeration-induced clustering may lead to increased abrasive interactions, posing challenges for practical applications. Therefore, future studies should focus on long-term ageing behaviour, the scalability of the functionalization process, and system-level performance under industrial operating conditions.

## Figures and Tables

**Table 1:** Rolling test parameters and experimental notation.

Rolling Parameter (L/R)								
Lubrication Condition (L)	Indicator	D	L	0.05G	0.1G	0.2G	0.4G	0.6G
	Meaning	Dry	Neat lubricant	0.05 wt% GNPs doped	0.1 wt% GNPs doped	0.2 wt% GNPs doped	0.4 wt% GNPs doped	0.6 wt% GNPs doped
Reduction Ratio (R)	Indicator	3				9		
	Meaning	3% reduction ratio				9% reduction ratio		

**Table 2:** Oxidation onset temperature (Tₒ) of mineral lubricant as a function of GNPs content.

Sample	Oxidation Onset Temperature (T_o_) (°C)
Neat	205.3
0.05%	207.2
0.1%	206.4
0.2%	200.1
0.4%	197.9
0.6%	176.8

## Data Availability

The original contributions presented in this study are included in the article. Further inquiries can be directed to the corresponding author.
